# NogoA Neutralization Promotes Axonal Restoration After White Matter Injury In Subcortical Stroke

**DOI:** 10.1038/s41598-017-09705-0

**Published:** 2017-08-25

**Authors:** Laura Otero-Ortega, Mari Carmen Gómez-de Frutos, Fernando Laso-García, Alba Sánchez-Gonzalo, Arturo Martínez-Arroyo, Exuperio Díez-Tejedor, María Gutiérrez-Fernández

**Affiliations:** Neuroscience and Cerebrovascular Research Laboratory, Department of Neurology and Stroke Center, La Paz University Hospital, Neuroscience Area of IdiPAZ Health Research Institute, Autonomous University of Madrid, Madrid, Spain

## Abstract

Blocking axonal growth inhibitor NogoA has been of great interest for promoting axonal recovery from neurological diseases. The present study investigates the therapeutic effects of blocking NogoA, inducing functional recovery and promoting white matter repair in an experimental animal model of stroke. Adult male rats were subjected to white matter injury by subcortical ischemic stroke. Twenty-four hours after surgery, 250 ug of anti-NogoA or anti-IgG-1 were administered through the tail vein. The quantity of NogoA protein was determined by immunohistochemistry in the brain and peripheral organs. In addition, functional status, lesion size, fiber tract integrity, axonal sprouting and white matter repair markers were analyzed. Moreover, an *in vitro* study was performed in order to strengthen the results obtained *in vivo*. A lower quantity of NogoA protein was found in the brain and peripheral organs of the animals that received anti-NogoA treatment. The animals receiving anti-NogoA treatment showed significantly better results in terms of functional recovery, fiber tract integrity, axonal sprouting and white matter repair markers compared with the control group at 28 days. White matter integrity was in part restored by antibody-mediated inhibition of NogoA administration in those animals that were subjected to an axonal injury by subcortical stroke. This white matter restoration triggered functional recovery.

## Introduction

After decades of research focused on a treatment for cortical infarcts in experimental models in which the gray matter is most affected, a few translational studies are beginning to highlight the importance of considering the white matter component after stroke. Human white matter makes up approximately half of the forebrain volume^1^. White matter is highly vulnerable to ischemia and has been directly associated with the development of clinical symptoms such as sensory and motor disturbances, paralysis, hemiparesis, psychiatric disorders and cognitive impairments. Despite the variety of scientific advances to promote white matter repair, there is currently no efficient treatment to recover all of these functional and cognitive deficits.

The ability of the adult brain to repair white matter lesions after damage is limited. This lack of ability might be due, at least in part, to molecules that limit axonal growth, such as myelin-associated inhibitors. Myelin-associated inhibitors are proteins mainly expressed by oligodendrocytes, which are thought to limit axon growth *in vivo* after central nervous system (CNS) injury. One of the most well-characterized myelin-associated inhibitors is neurite outgrowth inhibitor (NogoA)^2, 3^, which interacts with a neuronal Nogo-66 receptor 1 and plays an inhibitory role in axonal regeneration, repair and collateral sprouting in the CNS^2–7^. Therefore, blocking NogoA has been of great interest in recent years because it might promote axonal regeneration after white matter damage. In this sense, antibodies that target NogoA have been shown to promote neurite maturation *in vitro*, axonal growth and functional recovery after cortical stroke^8–10^ and could also be a good therapeutic treatment for white matter ischemic damage.

Thus, NogoA neutralization by intravenous administration of its antibody could enhance white matter repair processes after subcortical stroke in rats. Consequently, the aim of this study was to investigate the possible therapeutic effects of anti-NogoA in an experimental animal model of subcortical stroke.

## Results

### *In vitro* studies

#### Effectiveness of anti-NogoA in blocking NogoA

**Figure 1:**
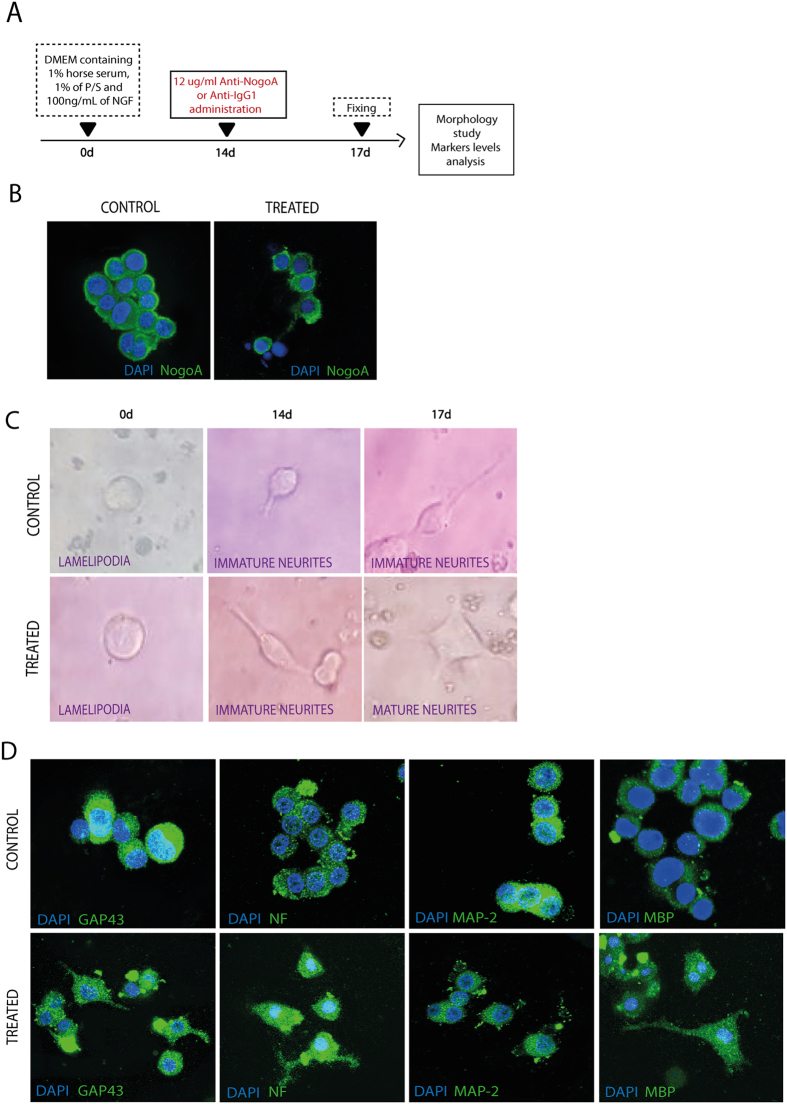
*In vitro* study of anti-NogoA administration. (**A**) Schematic experimental protocol of the *in vitro* analysis. (**B**) Anti-NogoA antibody treatment decreased NogoA protein expression compared with Anti-IgG antibody. (**C**) Axonal growth was observed in PC12-differentiated cells after anti-NogoA antibody administration using phase-contrast microscopy. (**D**) Immunocytochemistry images of PC12 cells showed that anti-NogoA antibody treatment increased expression of GAP-43, NF, MAP-2 and MBP markers in PC12 cells compared with Anti-IgG antibody administration. Abbreviations: GAP-43, growth associated protein 43; NF, neurofilament; MAP-2, microtubule-associated protein 2; MBP, myelin basic protein.

Following the steps of the schematic shown in Fig. 1A, the expression of protein NogoA was decreased in PC12 cells after 72 h of incubation with anti-NogoA compared with those cells that did not receive the antibody (Fig. 1B).

#### Axonal sprouting

PC12 cells were incubated with anti-NogoA antibody for 72 h. After that, PC12 cells changed their morphology, increasing neurite maturation compared with PC12, which did not receive anti-NogoA treatment (Fig. 1C).

#### White matter-associated marker expression

The levels of the expression of white matter-associated markers (growth-associated protein 43 [GAP-43], Neurofilament NF, microtubule-associated protein 2 [MAP-2] and myelin basic protein [MBP]) were increased 72 h after treatment with anti-NogoA in PC12 cells (Fig. 1D).

### *In vivo* studies

#### Effectiveness of anti-NogoA in terms of protein neutralization

**Figure 2:**
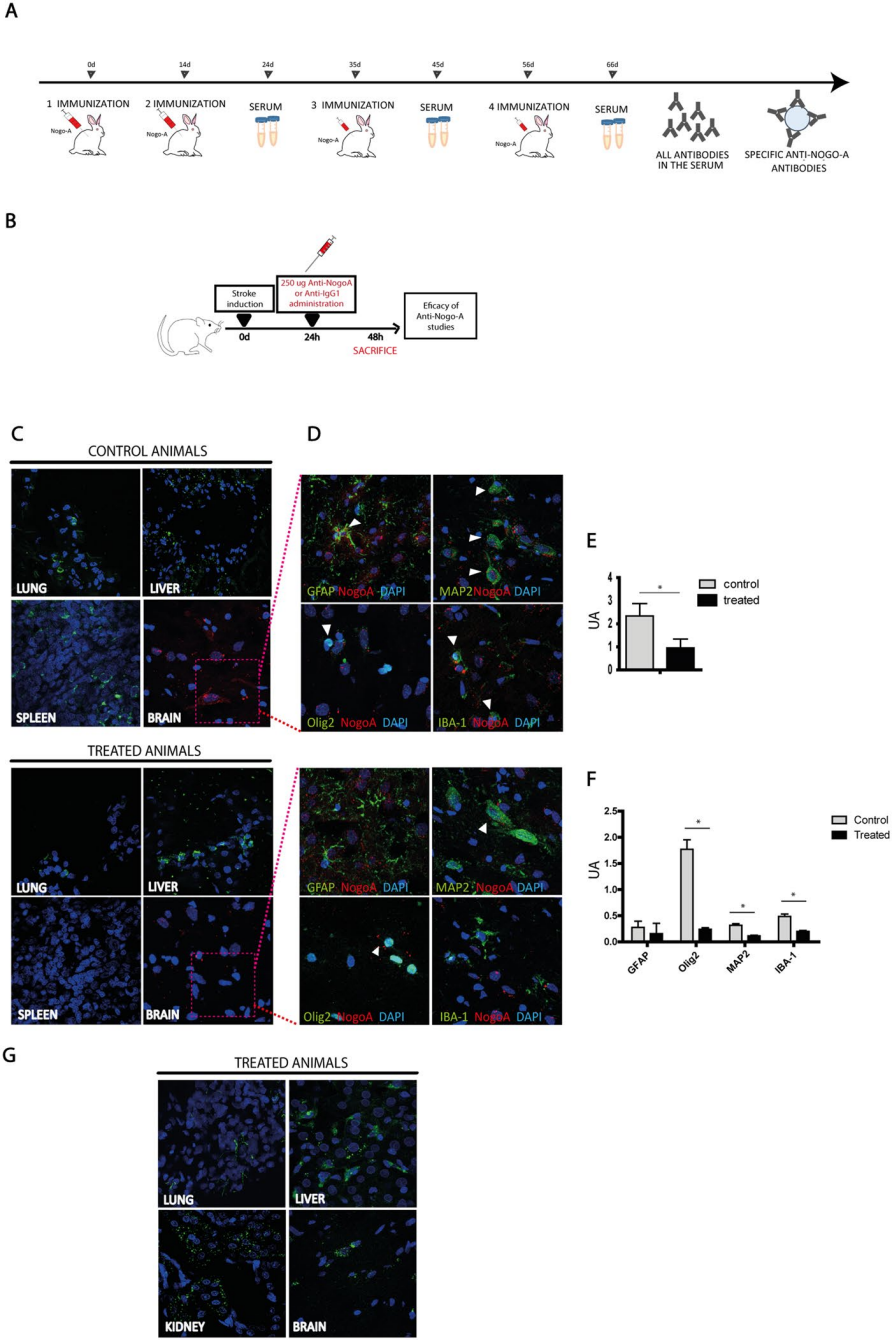
Anti-NogoA as antibody. (**A**) Production process of the anti-NogoA antibody, indicating time of immunization, serum collection and isolation of the Anti-NogoA antibody. (**B**) Experimental protocol schematic. Rats were subjected to a subcortical stroke by endothelin I injection. Twenty-four hours later, the rats received treatment (anti-IgG or anti-NogoA antibodies). At 48 h, histological studies to determine the effectiveness of the antibody were performed. (**C**) Immunofluorescence showing the presence of NogoA protein in the peripheral organs (lung, liver and spleen) and the brain in both the control and the treated groups 24 h after treatment. (**D**) Colocalization of the NogoA protein was shown with GFAP, Olig-2, MAP-2 and IBA-1 in brain samples. (**E**) Quantification of immunofluorescence showed a decrease in NogoA protein in the animals that received anti-NogoA antibody. (**F**) Quantification of the colocalization of the NogoA protein with GFAP, Olig-2, MAP-2 and IBA-1. Abbreviations: GFAP, glial fibrillary acidic protein; Olig-2, oligodendrocyte transcription factor-2; MAP-2 microtubule-associated protein 2; IBA-1, ionized calcium-binding adapter molecule 1. (**G**) Biodistribution of Anti-NogoA antibody showed the antibodies (green) in the lung, liver, kidney and brain by immunofluorescence at 24 hours after i.v. administration.

For this experiment, the steps shown in Fig. 2A were followed. An immunofluorescence analysis showed that NogoA protein was found in the lung, liver and spleen, as well as in the brain (Fig. 2C). Quantification of immunofluorescence showed that the animals that received anti-NogoA antibody had lower levels of NogoA protein in the brain compared with controls (0.82 ± 0.32 ua vs. 2.34 ± 0.53 ua, respectively) (p < 0.05) (Fig. 2E). We also found colabeling between NogoA protein and glial fibrillary acidic protein (GFAP), MAP-2, oligodendrocyte transcription factor-2 (Olig-2) and ionized calcium-binding adapter molecule 1 (IBA-1) at 24 h after anti-NogoA administration (Fig. 2D). The levels of NogoA protein were lower in the control animals compared with the treated animals in cells expressing MAP-2 (0.31 ± 0.02 ua vs. 0.11 ± 0.01 ua, respectively) (p < 0.05), Olig-2 (1.76 ± 0.18 ua vs. 0.24 ± 0.03 ua, respectively) (p < 0.05) and IBA-1 (0.48 ± 0.04 ua vs. 0.20 ± 0.018 ua, respectively) (p<0.05), but not in the cells expressing GFAP (0.27 ± 0.12 ua vs. 0.15 ± 0.19 ua, respectively) (p > 0.05) (Fig. 2F).

Moreover, anti-NogoA antibody biodistribution was analyzed 24 h after intravenous administration, and the antibodies were found in the brain and in the peripheral organs (lung, liver and kidney) (Fig. 2G).

### Functional recovery

**Figure 3:**
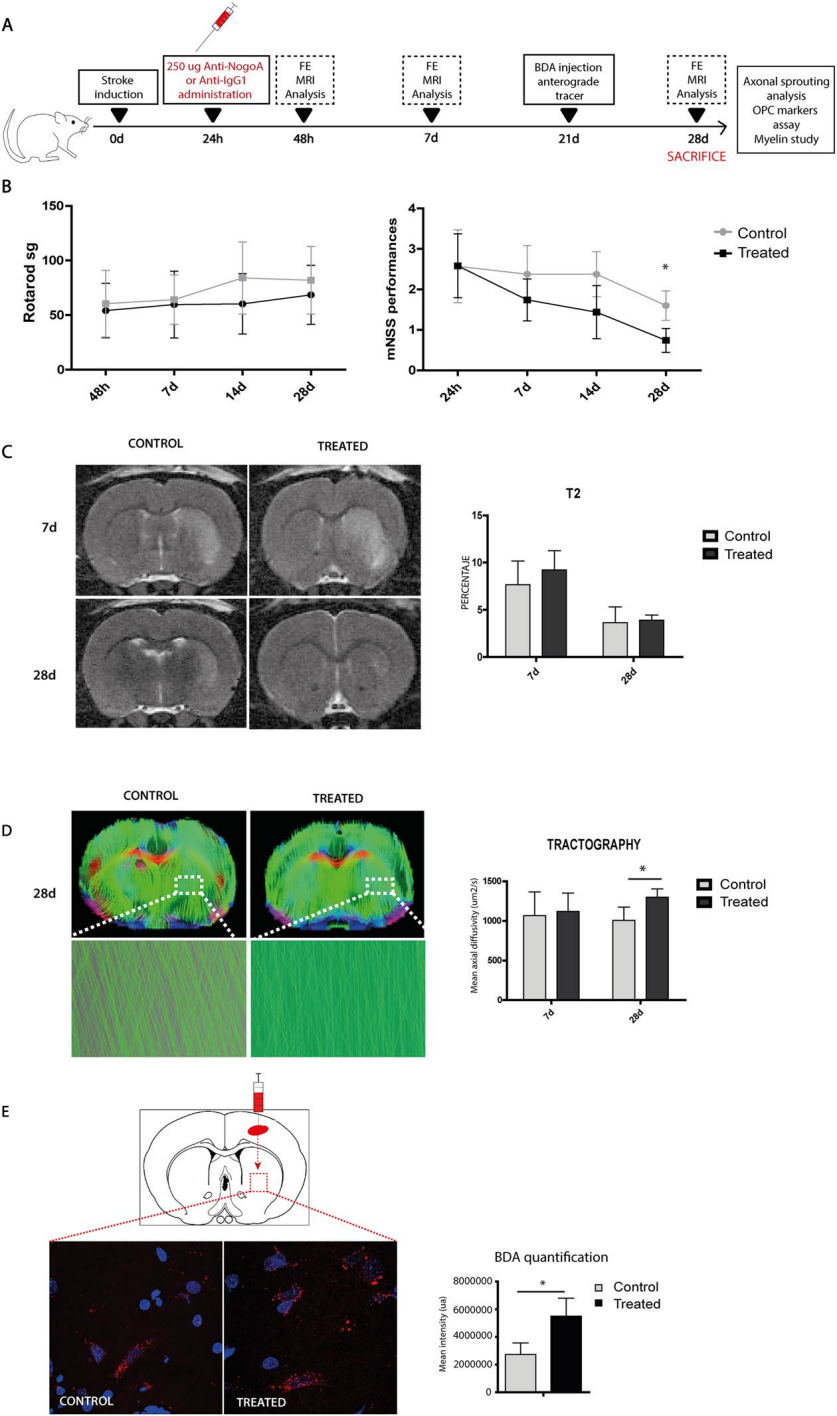
Improved functional outcome, increased fiber tract and axonal sprouting after anti-NogoA treatment in subcortical stroke. (**A**) Experimental protocol schematic. Rats were subjected to a subcortical stroke and 24 h later received treatment (anti-IgG or anti-NogoA antibodies). Then, 48 h after the stroke, on day 7 and 28, behavior and imaging studies were evaluated. Later, 21 d after stroke, BDA was injected as an anterograde tracer. At 28 d, histological studies were analyzed. (**B**) The rotarod test (left), the mNSS test (right). The mNSS performances were improved at 28 d in the treatment group compared with the controls (p < 0.05). Data are shown as mean ± SD, *p < 0.05; n = 10 animals per group. (**C**) Comparative image analysis of T2-weighted MRI at 7 d and 28 d (left) and quantitative analysis of T2 images (right). Quantitative analysis of tractography. Data are shown as mean ± SD, *p < 0.05, n = 4 animals. (**D**) Comparative image analysis of tractography. Detail of tractography image in the lesion is shown below at 28 d. Quantitative analysis of tractography. Data are shown as mean ± SD, *p < 0.05, n = 4 animals. (**E**) Representative photomicrographs of striatal neuronal projections labeled with the anatomical tracer BDA (left). Quantification of striatal cells that were projected from cortical cells labeled with BDA (right). Data are shown as mean ± SD, *p < 0.05, n = 4 animals. Abbreviations: BDA, biotinylated dextran amine.

For this experiment, the steps shown in Fig. 3A were followed. No significant differences were found in the functional outcome of the treated and control animals in the rotarod test (p˃ 0.05). However, 28 d after treatment with anti-NogoA, the animals showed significantly better performance on the modified neurological severity score (mNSS) test (0.74 points ± 0.24 points) compared with the control group (1.60 points ± 0.36 points) (p < 0.05) (Fig. 3B).

### Lesion size and tract connectivity

Lesion sizes of both the control and the treated groups were reduced at 7 to 28 d after stroke. However, we did not observe significant differences between groups either at 7 or at 28 d after stroke; (p > 0.05) (Fig. 3C).

The tractography analysis, however, showed significantly improved DTI performance in the treated animals (1297.74 µm^2^/s ± 106.41 µm^2^/s) compared with the controls (1008.61 µm^2^/s ± 165.33 µm^2^/s) 28 d after stroke (Fig. 3D).

### Axonal sprouting

Stroke was produced in the subcortical white matter of the striatum. This region contains fascicles of axons projecting from the overlying cortex. Motor cortical projections play an important role in motor function and movement. The anatomical anterograde neuronal tracer biotin dextran amine (BDA) was injected into the forelimb motor cortex 21 d after stroke, and the striatal axonal density was studied 7 d after the injection (28 d after stroke) to analyze changes in axonal sprouting after the treatment. A statistically significantly high mean intensity of BDA-labeled axons were observed in the striatum in the treated group compared with the control animals (5,497,276.06 ua ± 1,311,049.01 ua vs. 2,727,665.32 ua ± 839,278.50 ua) (p < 0.05). These results might indicate that anti-NogoA antibody treatment produces a significant axonal sprouting response from the ipsilateral cortex to the injured striatum (Fig. 3E).

### White matter-associated marker expression

**Figure 4:**
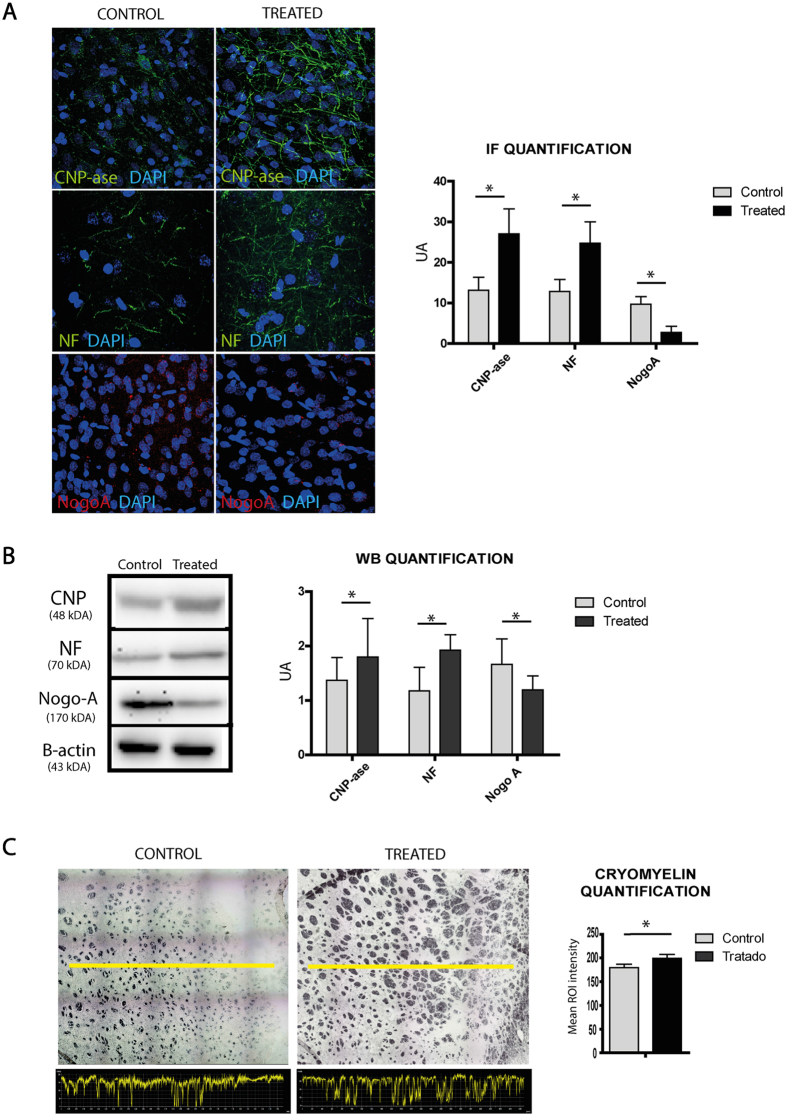
White matter-associated markers are enhanced after anti-NogoA therapy in a subcortical stroke model. (**A**) Immunofluorescence images of white matter repair-associated markers (CNPase, NF and NogoA) (left). Quantification of ROI of the immunofluorescence. Data are shown as mean ± SD, *p < 0.05, n = 4 animals. (**B**) Western blot images. The gel images are cropped. Quantification of WB. Data are shown as mean ± SD, *p < 0.05, n = 4 animals. (**C**) Morphological study by CryoMyelin staining identified the zone of the lesion as an area of white matter injury located in the subcortical zone, showing restored myelinated axons in the treated animals. The yellow line indicates a representative longitudinal profile of pixel intensity (left). Quantification of the white color along the yellow line (below) and quantification of the mean ROI intensity of the CryoMyelin staining (right). Data are shown as mean ± SD, scale bars = 20 µm, *p < 0.05, n = 4 animals, 5 sections each per group. Abbreviations: CNPase: 2′,3′-cyclic-nucleotide 3′-phosphodiesterase; NF: neurofilament; WB: western blot.

The levels of white matter-associated markers and NogoA protein were analyzed in the lesion area 28 d after treatment (Fig. 4). Immunofluorescence analysis showed significantly higher levels of 2′,3′-cyclic-nucleotide 3′-phosphodiesterase (CNPase) (27.02 ± 6.21 vs. 13.14 ± 3.21, respectively) (p < 0.05) and neurofilament (NF) marker (24.7 ± 5.28 vs. 12.80 ± 2.98, respectively) (p < 0.05) in the treated animals compared with the controls. Regarding NogoA, we found lower levels of this protein in the treated group (2.80 ± 1.49) compared with the controls (9.67 ± 1.87) (p < 0.05) (Fig. 4A). These results were confirmed by western blot analysis. There was a significant increase in CNPase marker levels in the treated animals (1.79 ± 0.71) compared with the controls (1.39 ± 0.42) (p < 0.05), and NF was higher in the treated animals (1.92 ± 0.28) than in the controls (1.17 ± 0.43) (p < 0.05). We also found lower levels of NogoA marker in the treated group (1.19 ± 0.25) than in the control animals (1.66 ± 0.46) (p < 0.05) (Fig. 4B).

### Myelin restoration

A morphological study using a HitoCryoMyelin staining kit identified the lesion zone in all the animals as a subcortical infarct affecting the white matter, with more myelinated axons in the treated animals (199.19 ± 8.17) compared with the control group (179.56 ± 7.31) (p < 0.05) (Fig. 4C).

## Discussion

Blood supply disruption in stroke compromises not only some neurons but also whole axons and fibers, and therefore, brain connectivity. However, white matter injury and the mechanisms of nerve fiber (axon and myelin) repair have seldom been investigated in translational stroke research^11^. The high incidence of such damage motivates the search for an effective therapy to enhance the mechanisms underlying the repair of damaged nerve fibers after any kind of stroke. White matter repair after stroke can lead to processes that include the activation of neuronal molecular mechanisms of growth, extrinsic growth promoting factors and axon guidance signals in the tissue. Almost nothing is known about the optimal details of the various processes; we only know that pharmacological treatments can modify these neuronal processes. Finding a therapy in accordance with these processes (in terms of time window and doses) would be the key for a correct recovery after stroke. In this sense, myelin-associated inhibitors such us NogoA are proteins that inhibit neurite growth in adults and are thought to limit axon growth and play an inhibitory role in axonal regeneration, repair and collateral sprouting *in vivo* after CNS injury^4–6, 12, 13^. Thus, antibodies that target NogoA might promote axonal growth. In this study, we observed that NogoA neutralization enhanced axonal repair, tract connectivity, oligodendrogenesis and myelin formation in the striatum as well as motor functional recovery after white matter stroke in an animal model. These findings were correlated with improved motor skill recovery upon blockade of NogoA function.

Many studies have used anti-NogoA therapy as a treatment after various neurological disorders such as stroke. Most previous studies have administered NogoA antibody by intracerebral^8, 14, 15^ or intrathecal routes^14–18^, using osmotic minipumps. However, to be more appropriate for translation of this treatment to clinical applications, we chose the intravenous route, which is a less invasive route previously described by another study on spinal cord injury as safe and effective^19^.

The delivery of anti-NogoA antibodies could allow their union with the protein and neutralize their negative effect on neurite and axonal outgrowth after damage. In this sense, it is important to identify in which organ (brain or other peripheral organs) the antibody anti-NogoA is acting. NogoA is enriched on the surface of oligodendrocytes in the outermost and innermost axonal myelin membrane. It is important to note that new findings showed that NogoA is not only expressed on oligodendrocytes but also in various brain areas and in several types of neurons and glial cells^20^. In our study, we observed a lower expression of NogoA protein in the brain and peripheral organs (lung, liver and spleen) compared with control animals. We also found a lower expression of NogoA protein in cells expressing Olig-2, MAP-2 and IBA-1, but not in cells expressing GFAP. These findings suggest that the anti-NogoA antibody affects not only oligodendrocytes, but also neurons, microglia and astrocytes. Moreover, the NogoA protein was also analyzed at 28 d after treatment, and the levels of this protein were lower in the treated animals compared with the controls. These results indicate that anti-NogoA injection decreases NogoA levels at least 28 d after treatment. These results are consistent with those found in a biodistribution analysis, in which anti-Nogo-A antibodies were found in the brain and in the peripheral organs (lung, liver and kidney), where the NogoA protein expression was decreased.

In a translational study, it is important to analyze whether anti-NogoA injections act on the motor dysfunctions characteristic of subcortical stroke. Previous authors found the rotarod test^21^ and the mNSS test^22^ to be effective in assessing the motor deficit associated primarily with subcortical stroke. In this study, anti-NogoA antibody treatment induced a significant improvement in functional recovery that was particularly notable at 28 d after treatment when compared with the controls. Although there are no previous studies that have administered anti-NogoA in a subcortical stroke model, our results are consistent with previous data showing that anti-NogoA immunotherapy improved functional recovery in an experimental animal model of middle cerebral artery occlusion^8, 9, 16, 18, 23^, and even in post-acute lesions^14^.

Some experimental studies show that a change in the reduction of lesion volume is not important to determining the severity of a cerebral infarct. In fact, infarct volume did not correlate with functional recovery when the neurovascular unit was integrated^24^. In our study, no reduction in the lesion size was observed on T2 images in animals treated with anti-NogoA immunotherapy compared with the control group at 24 h or 28 d. However, these results are consistent with previous studies that showed that this treatment did not reduce lesion size in an experimental animal model of cortical stroke^9, 17, 18, 25^.

Stroke triggers axonal sprouting in the cortical areas adjacent to the infarct. This sprouting can be detected with anatomical mapping of cortical circuits as early as 3 weeks after stroke^26^. Axonal sprouting in the adjacent cortex after stroke occurs in the tissue immediately bordering the infarct and in motor, somatosensory and premotor areas distant from the infarct^27^. To map the fascicles of axons projecting from the overlying motor cortex that were growing after anti-NogoA treatment, neurons were anterogradely labeled using a BDA injection into the cortex 3 weeks after the stroke. Anterograde tracing with BDA demonstrated descending projections from the intact cortex. A statistically significantly high number of BDA-labeled axons were shown in the striatum of the animals that received treatment with anti-NogoA antibody compared with the control animals. These results might indicate that blocking NogoA produces a significant axonal sprouting response from the ipsilateral cortex to the injured striatum. These results correlate with those studies examining NF, given we observed that the levels of this marker were also higher in the treated animals compared with the control animals at 28 d after stroke. This result agrees with a previous study using retrograde tracing that showed a 2- to 3-fold increase in labeled neurons projecting to both sides of the spinal cord in the anti-NogoA-treated animals^28^. These results are consistent with previous data showing that the anti-NogoA antibody promotes axonal growth after various types of CNS injury^8, 9, 29^. Moreover, three various anti-NogoA antibodies have proven efficient in enhancing axonal regeneration and outgrowth both *in vitro* and *in vivo* ^30^. Another study revealed overexpression of neuronal GAP-43, an intrinsic determinant of neuronal plasticity and a molecular indicator of axonal plasticity in the CNS^31^. Moreover, consistent with these results, other authors have demonstrated that a blockade of NogoA leads to greater synaptic plasticity and modification^32^. These results suggest that functional recovery at 28 d could be related to the process by which restructured axons, which had previously been injured, recover axon integrity and tract connectivity.

In particular, myelin-associated inhibitors are proteins thought to limit axon growth and collateral sprouting^2–6, 12, 13, 33^. Thus, antibodies that target NogoA might promote tract integrity. To analyze the fiber tract integrity after antibody anti-NogoA, we performed *in vivo* DTI tractography, which showed that tract thickness was recovered at 28 d after treatment compared with controls. These results suggest that blocking NogoA could promote not only axonal growth but also track integrity and connectivity. This treatment could be involved in the process by which restructured axons, which had previously been compromised and demyelinated, recover not only their proper structure but also tract connectivity.

NogoA is predominantly expressed by oligodendrocytes^13^. After white matter stroke, damage to oligodendrocytes leads to white matter dysfunction through the loss of myelin. Then, enhanced proliferation, migration and differentiation of oligodendrocyte progenitor cells (OPCs) are observed in damaged and recovering white matter regions^34^. To assess whether NogoA blockage enhances oligodendrogenesis, we measured CNPase (a marker related to immature oligodendrocytes) by immunofluorescence and western blot. In our study, the levels of CNPase marker were higher in the treated animals at 28 d than in the control group.

Not only axons and oligodendrogenesis are important in the process of white matter repair. In addition, the myelin sheath allows the transmission of nerve impulses over relatively long distances, which is a process necessary for whole brain function^35^. A cryomyelin study showed an increase in myelin in those animals that received treatment with anti-NogoA compared with the control group.

*In vitro* experiments help to strengthen results found in an *in vivo* model. In agreement with previously published studies^10, 36, 37^, PC12 cells have differentiated neurites in the presence of nerve growth factor (NGF). When comparing the response of the anti-NogoA antibody group to the NGF-treated cells, the treated cells appeared to have a higher percentage of immature and mature neurites. The enhancement of neurite maturation appeared to be directly correlated with the presence of anti-NogoA at a concentration of 12 µg/ml. Our results demonstrated that although the NGF-treated cells exhibited immature neurites, the anti-NogoA antibody cells exhibited many more mature neurites. These results agree with other studies in which the pretreatment of PC12 cells with the anti-NogoA antibody was effective in blocking the inhibitory effects of the NogoA peptide in terms of neurite length, as observed for the NGF-treated group^10^. Moreover, we analyzed whether the anti-NogoA antibody was able to decrease NogoA protein levels *in vitro*. The results of this study showed a lower expression of the NogoA protein in PC12 cells treated with the anti-NogoA antibody compared with the control culture. Moreover, our results showed that the anti-NogoA antibody triggered an increase in neural marker expression (GAP-43, NF, MAP-2 and MBP) on PC12 cultures.

In summary, the enhancement of behavioral recovery in a variety of sensory-motor tasks, as well as anatomical evidence of fiber growth, tract connectivity and oligodendrogenesis, have been reported after anti-NogoA antibody administration in a subcortical stroke animal model.

## Materials and Methods

### *In vitro* studies

#### Effectiveness of anti-NogoA blocking NogoA

To study whether the anti-NogoA antibody induced a block of NogoA expression, an *in vitro* study using PC12 cells was performed. PC12 cells (Sigma-Aldrich, catalogue number 88022401) were maintained in Dulbecco’s Modified Eagle’s Medium (DMEM; Invitrogen) high glucose, supplemented with 10% horse serum (Sigma-Aldrich), 10% fetal bovine serum (Biowest) and 1% penicillin/streptomycin (P/S) at 5% CO2, at 37 °C. For the NGF treatment (NGF-7S, Sigma-Aldrich) to induce neurite and axonal outgrowth in PC12 cells, they were plated onto poly-l-lysine (Sigma-Aldrich) coated dishes and maintained in DMEM containing 1% horse serum, 1% P/S and 100 ng/mL NGF. After 14 d, anti-NogoA antibody (12 µg/mL, Immunostep S.L.) was incubated with half the cultures for 72 h (Fig. 1A). Next, the expression of NogoA protein was determined by immunofluorescence using the NogoA antibody (1:100, Abcam). A secondary antibody for immunofluorescence was goat anti-mouse Alexa Fluor 488. The slices were examined using a LEICA TCS SPE spectral confocal microscope (Leica Microsystems, Heidelberg, Germany), and the confocal images were analyzed using LEICA software LAS AF, version 2.0.1, Build 2043.

#### Axonal sprouting

After 72 h of incubation with anti-NogoA antibody, changes in morphology and neurite outgrowth of PC12 cells were studied using a Nikon Eclipse-Ti inverted microscope and NIS-elements software.

#### White matter-associated marker expression

Expression of neural markers after anti-NogoA treatment on PC12 cells was measured by immunocytochemistry, using the following antibodies: GAP-43 (1:500, Abcam), NF (1:100, Dako), MAP-2 (1:1000, Millipore) and MBP (1:100, Abcam). Secondary antibodies for immunofluorescence were goat anti-mouse Alexa Fluor 488 and anti-rabbit Alexa Fluor 488 (1:750, Invitrogen). The slices were examined using a LEICA TCS SPE spectral confocal microscope (Leica Microsystems) and the confocal images were analyzed using LEICA software LAS AF, version 2.0.1, Build 2043.

### *In vivo* studies

#### Ethics statement

All the procedures were performed in accordance with stroke therapy academic industry roundtable^38, 39^ and RIGOR^40^ relevant guideline recommendations in terms of randomization, blinding and statistical powering. All the experimental protocols were approved by the medical school’s Ethics Committee for the Care and Use of Animals in Research of La Paz University Hospital, according to Spanish and European Union rules (86/609/CEE and RD53/2013). This committee includes endpoint criteria. All the animals received a postsurgery injection of meloxicam as analgesia, and we have a protocol for the daily supervision of animals. If any animals show pain symptoms, they again receive meloxicam (2 mg/kg) as analgesia.

#### Animals and surgical procedure

To provoke white matter injury, a subcortical ischemic stroke was induced in male Sprague-Dawley rats (200–250 g, Charles River Laboratories). Anesthesia was induced using an anesthetic chamber with 5% isoflurane in a 5-L/min oxygen flow, and the animals were maintained using a face mask with 3% isoflurane in a 2-L/min oxygen flow. In all the animals, the femoral artery was cannulated during surgery and induction of cerebral ischemia. The cannulation allowed for continuous monitoring of physiological parameters including blood glucose levels, blood gases and blood pressure (Omicron ALTEA Monitor; RGB Medical Devices, Madrid, Spain). After an intraperitoneal injection of meloxicam (2 mg/kg), the animals were placed in a stereotactic frame. A craniotomy was performed adjacent to the bregma and intrastriatal white matter injury was induced by injection of 1 µL of endothelin-1 (ET-1; Calbiochem) (0.25 µg/µL) for subcortical ischemic stroke. Stereotaxic coordinates of the injection site with respect to the bregma were as follows: 0.04 mm posterior, 0.35 mm lateral and 0.6 mm ventral, as previously described^21, 22^.

#### Anti-NogoA formation

On day 0, NogoA protein and the appropriate adjuvants were injected into the host, which were recognized by the immune system as foreign and were targeted by antibodies for immune blockage. All antigen-presenting cells internalize antigens by endocytosis or phagocytosis. Recognition of antigens induces the release of stimulatory signaling, thus activating B cells. Activated B cells divide to create both memory B-cell and plasma cell populations. Plasma cells secrete antibodies into the serum for immune recognition, whereas memory B-cells persist for longer, thus providing a quick response to secondary exposure from the same antigen. This process is termed immunization. Fourteen days after the first immunization, the second immunization was performed, again injecting the NogoA protein and the appropriate adjuvants. At 24 d, serum from the rabbit was collected. At 35 d after first immunization, a third immunization was performed, and serum was collected at 45 d. Finally, a fourth immunization was performed at 56 d, and serum was collected at 66 d. Measuring the antibody levels by ELISA during the immunization process is required to evaluate progress. Because many antigen-specific antibodies are secreted by multiple activated B-cells, anti-NogoA antibodies need to be isolated from the other antibodies (Fig. 2A).

#### Experimental groups

A total of 33 male Sprague-Dawley rats (8–9 weeks old, weighing 200–250 g) were used in this study. The study groups were as follows: (I) sham-operated group (n = 5); (II) control group: subcortical ischemic stroke + 250 µg of rat anti-IgG (Immunostep S.L.) diluted in 500 µl of intravenous PBS (n = 10); (III) treated group: subcortical ischemic stroke + 250 µg of rat anti-NogoA (polyclonal, Immunostep S.L.) diluted in 500 µl of intravenous PBS (n = 10).

Additionally, 8 animals were used in order to test the effectiveness of the anti-NogoA injection to block rat NogoA protein. All the animals were subjected to stroke, and 24 h later, 4 animals received anti-NogoA and 4 animals received anti-IgG. These animals were euthanized 24 h after treatment. The brains and peripheral organs (liver, lung, kidney and spleen) were processed to perform immunofluorescence to analyze the levels of the NogoA protein in the various tissues (Fig. 2B).

Treatment was administered via the tail vein 24 h after surgery, and the remaining animals were euthanized at 28 d (Fig. 3A). Five rats were excluded from the study because 2 rats died during surgery and 3 rats died during the magnetic resonance procedure (2 from the control group and 1 from the treated group).

#### Effectiveness of anti-NogoA in terms of protein neutralization

To study whether the anti-NogoA antibody induced a block of NogoA protein *in vivo*, the antibody was intravenously administered 24 h after the animals suffered a stroke. The expression of NogoA protein was determined by immunofluorescence using NogoA antibody (1:100, Abcam) in the brain and in the peripheral organs (lung, liver, kidney and spleen) 24 h after anti-NogoA administration. To determine in which type of cells the expression of NogoA was blocked, colabeling was performed with the NogoA antibody (1:100, Abcam) and GFAP (1:400, Chemicon), MAP-2 (1:1000, Milipore), Olig-2 (1:500, Millipore) and IBA-1 (1:1000, Milipore) in the brain samples. Secondary antibodies for immunofluorescence were goat anti-mouse Alexa Fluor 488 and anti-rabbit Alexa Fluor 488/594 (1:750, Invitrogen). The slices were examined using a LEICA TCS SPE spectral confocal microscope (Leica Microsystems), and the confocal images were analyzed using LEICA software LAS AF, version 2.0.1, Build 2043. Moreover, the biodistribution of the anti-NogoA antibody was studied after incubating the slides of the brain and peripheral organs in secondary antibody anti-rabbit Alexa Fluor 488 (1:750, Invitrogen).

#### Functional evaluation scales

Functional evaluations were performed on all the animals by a blinded observer before surgery and after 48 h, 7 d, 14 d and 28 d. Motor performance was evaluated using the rotarod test and the mNSS. The rotarod test measured the time to falling from a rotating cylinder^21^. The mNSS test was used to measure sensory and motor deficits; it is a composite of motor, sensory, balance and reflex tests, and neurological function is graded on a scale of 0 to 18. A score of 0 is associated with normal neurological function, and a score of 18 represents the maximum functional deficit. For the severity scores of injury, 1 point is awarded for a specific abnormal behavior or for the lack of a tested reflex; thus, the more injured the animals, the higher the score on the test^41–43^. The researchers recording the data were entirely blinded to the experimental groups.

#### *In vivo* magnetic resonance imaging

Lesion sizes were analyzed after 7 d and 28 d by magnetic resonance imaging (MRI) using a 7-Tesla horizontal bore magnet (Bruker PharmaScan, Ettlingen, Germany) and T2-weighted images. The lesion area was expressed as a percentage of the contralateral hemisphere. For the tractography analysis, a diffusion-weighted MRI was acquired on a Bruker PharmaScan system (Bruker Medical Gmbh, Ettlingen, Germany) using a 7.0-T horizontal-bore superconducting magnet, equipped with a 1 H circular polarized volume coil with inner diameter of 40 mm and a Bruker gradient insert of 90-mm diameter (maximum intensity 30 G/cm). The animals were anesthetized with a 2% isoflurane-oxygen mixture in an induction chamber, and the flow of anesthetic gas was continuously regulated to maintain a breathing rate of 50 + /− 20 bpm. The animals’ temperature was maintained at approximately 37 °C with a circulating warm water blanket. The physiological state of the rats was monitored using a monitoring system by SA instruments (Stony Brook, NY) that controlled the respiratory rate and body temperature. Diffusion tensor data were acquired with a spin echo single shot echo planar imaging (EPI) pulse sequence using the following parameters: TR/TE 8000/35 ms; a signal average of 12, 30 noncollinear diffusion gradient scheme with a diffusion weighting b = 500 s/mm^2^ and b = 1000 s/mm^2^, 18 slices with a slice thickness of 1.5 mm without a gap, field of view 35 × 35 mm. Total imaging time was 1 h 44 min. All the EPI data were acquired with a single shot EPI sequence, 96 × 96 matrix, and zero filled-in k space to construct a 128 × 128 image matrix. Fractional anisotropy, mean diffusivity, trace and the eigenvalues and eigenvector maps were calculated with a homemade software application written in Matlab (R2007a); the 3D fiber tract map was created using MedINRIA DTI Track software, as previously described^41, 43^. The researchers recording the data were entirely blinded to the experimental groups.

#### Mapping of motor cortex connections

Changes in axonal sprouting after treatment were assessed using an intracortical injection of the anterograde neuronal tracer BDA (10000 MW, Life Technologies, Grand Island, NY, USA). The stereotaxic coordinates of the injection site with respect to the bregma were as follows: 0.04 mm posterior, 0.35 mm lateral and 0.16 mm ventral. The rat cortexes were injected with BDA at 21 d after stroke and they were euthanized 28 d after stroke. All the brains were fixed, frozen and coronally sectioned at 40 µm. The sections were visualized using a confocal microscope (Leica Microsystems). Mean intensity of striatal cells labeled with BDA was quantified using 4 animals, with 10 sections from each animal per group. The researchers recording the data were entirely blinded to the experimental groups.

#### Immunohistochemistry, immunofluorescence and western blot analyses

Frozen sections were stained using the CryoMyelin Kit (Hitobiotech), which allows sensitive localization and visualization of myelin fibers. The mean intensity of myelin staining in the region of interest was quantified using a Nikon Eclipse-Ti inverted microscope and NIS-elements software. The lesion area was studied in detail using immunofluorescence and western blot analyses. The various white matter-associated antibodies used for immunofluorescence and western blot analyses were CNPase (1:500, Sigma-Aldrich), NF (1:100, Dako) and NogoA (1:100, Abcam), followed by goat anti-mouse and anti-rabbit Alexa Fluor 488 (1:750, Invitrogen). For the western blot analysis, the units were normalized based on *Β*-actin (1:400, Sigma-Aldrich). To quantify the expression of white matter-associated markers, the mean fluorescence intensity was evaluated using a 40X objective lens (4 animals in each group, 5 sections in each animal per group, 10 different microscope fields). The experiments, images and quantification of the samples were performed by blinded observers, using the same microscope configurations, to eliminate bias due to background normalization.

### Statistical analysis

Results were expressed as mean ± standard deviation. The data were compared using the Kruskal-Wallis test followed by the Mann-Whitney test. Values of p < 0.05 were considered significant at a 95% confidence interval; the data were calculated using statistical SPSS 16 and GraphPad software. The power analysis showed that with nonparametric testing, at least 10 animals needed to be randomized to each group for a significance level of 5% (alpha) and a potency of 80% (1-beta) for functional evaluation.

## Electronic supplementary material

Supplementary info
