# Supplementary material for: NogoA Neutralization Promotes Axonal Restoration After White Matter Injury In Subcortical Stroke
Source: Sci Rep. 2017 Aug 25;7:9431. doi: 10.1038/s41598-017-09705-0 (PMC5573364; doi:10.1038/s41598-017-09705-0)
Supplement: Supplementary file 1 — Supplementary info [file 41598_2017_9705_MOESM1_ESM.pdf]

NogoA neutralization promotes axonal restoration after white matter injury insubcortical stroke

Laura Otero-Ortega; Mari Carmen Gómez-de Frutos*; Fernando Laso-García*; Alba Sánchez-Gonzalo; Arturo Martínez-Arroyo; Exuperio Díez- Tejedor§; María Gutiérrez- Fernández§

[1]Neuroscience and Cerebrovascular Research Laboratory, Department of Neurology and Stroke Center, La Paz University Hospital, Neuroscience Area of IdiPAZ Health Research Institute, Autonomous University of Madrid, Madrid, Spain

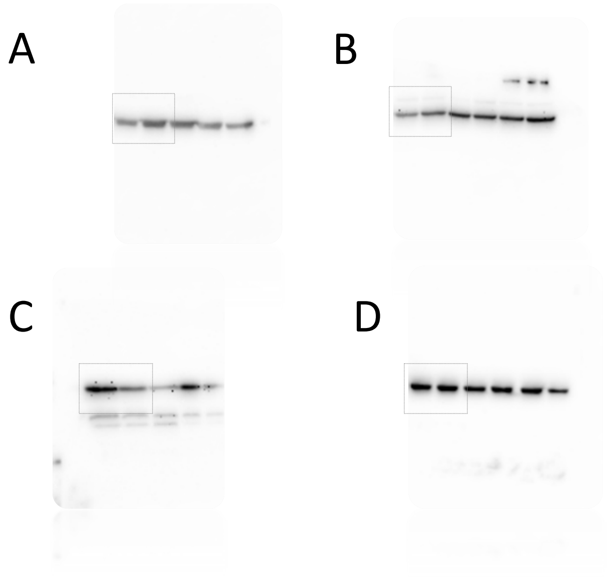

Figure S1: Uncropped and unprocessed original scans for all the blots in figure 4.
White matter repair associated markers [CNP-ase marker.(A), Neurofilament marker (B), NogoA marker (C)] were studied in the lesion zone of the brain by Western blot. D) B-actin marker was used as loading control in Westner blot . Stroked Squares represent the zone of the blot used for Figure 4.
